# Symptomatic Gastric Diverticulum in the Antrum: A Case Report

**DOI:** 10.7759/cureus.52449

**Published:** 2024-01-17

**Authors:** Mohammad N Kloub, Raed Atiyat, Muhammad Hussain, Byron Okwesili, Theodore A DaCosta

**Affiliations:** 1 Internal Medicine, Saint Michael's Medical Center, Newark, USA; 2 Gastroenterology and Hepatology, Saint Michael's Medical Center, Newark, USA

**Keywords:** diverticulosis, antrum, stomach, antral diverticulum, gastric diverticulum

## Abstract

Gastric diverticulum is an out-pouching that occurs in the gastric wall and, often, when found, is incidental and asymptomatic. While they do not occur commonly, gastric diverticula are located most commonly in the posterior wall of the fundus of the stomach, and their presence in the antrum, as appreciated in the case described below, is quite rare. We present a 65-year-old female who was found to have an antral gastric diverticulum on esophagogastroduodenoscopy (EGD). There have been a few reported cases in the literature of gastric diverticulum that occurred in the antrum. This case report will shed light on this rare pathology, focusing on the occurrence in the antrum.

## Introduction

Gastric diverticulum is a rare anatomic abnormality characterized by out-pouching within the stomach, mainly occurring in the posterior wall. Gastric diverticulum is similar to intestinal diverticulum but is considered the least common gastrointestinal diverticula and a rare pathology overall. As most individuals with gastric diverticulum are asymptomatic, it is usually discovered incidentally with a prevalence of 0.01-0.11% during an EGD [1,2]. Although most of the patients with gastric diverticulum are asymptomatic, patients can present with complaints of variable gastrointestinal symptoms ranging from epigastric pain to upper gastrointestinal bleeding, making the diagnosis more challenging [2]. Herein, we present a case report of a gastric diverticulum in a very unusual location.

## Case presentation

A 65-year-old female patient who had previously been diagnosed with hypertension presented complaining of generalized abdominal pain, more prominent in the epigastric region, that had been ongoing for two months. She characterized it as dull, non-radiating, more noticeable while she is resting on her back, and occasionally accompanied by nausea and vomiting of partially digested meals. The patient denied hematemesis, melena, hematochezia, fever, or diarrhea. She denies any history of alcohol intake or smoking.

Of note, the patient had a history of left hemicolectomy with colorectal anastomosis six months before presentation (02/24/2023) for large tubulovillous adenoma with high-grade dysplasia in the sigmoid that caused recurrent colitis.

On admission, the vitals were stable, the patient was comfortable, and not in acute distress. An abdominal exam revealed a healing surgical scar and a soft abdomen with mild tenderness in the epigastrium. Laboratory findings revealed a hemoglobin of 10.2 g/dl (normal value: 12-16 g/dl). The liver function test, kidney function test, lipase, amylase, and inflammatory markers were normal. The stool occult blood test was positive.

**Figure 1:**
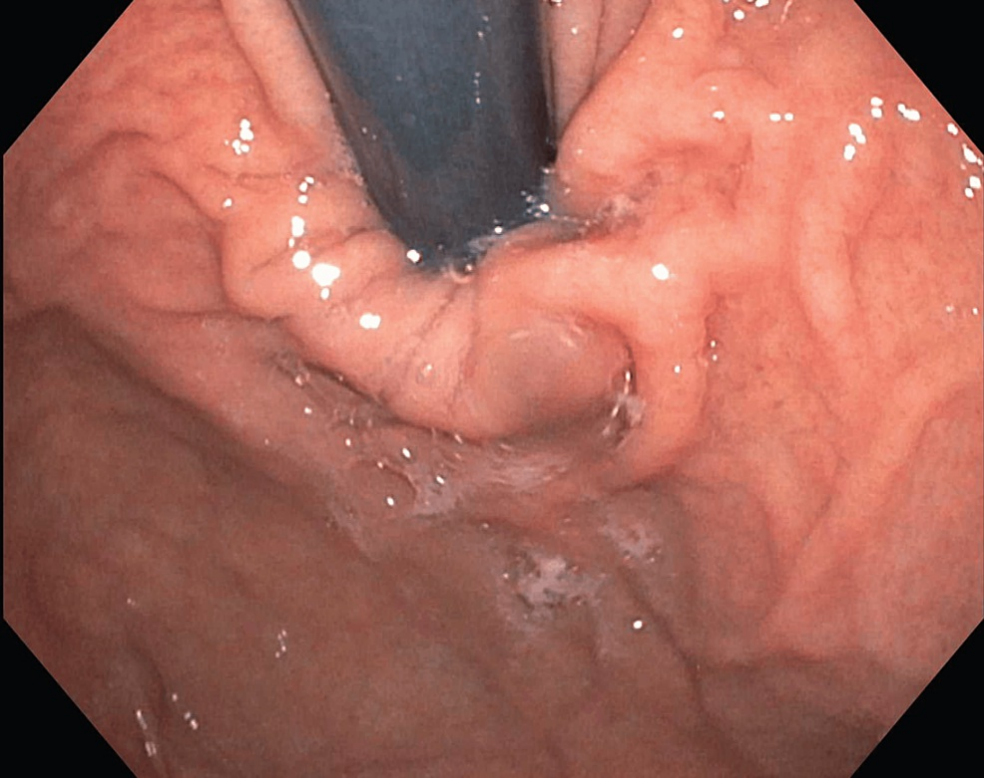
Scattered mild inflammation characterized by congestion (edema) and erythema in the gastric fundus

Colonoscopy revealed patent end-to-end colorectal anastomosis, with intact appearance and visible sutures. EGD revealed scattered mild inflammation characterized by congestion (edema) and erythema in the gastric fundus (Figure 1).

**Figure 2:**
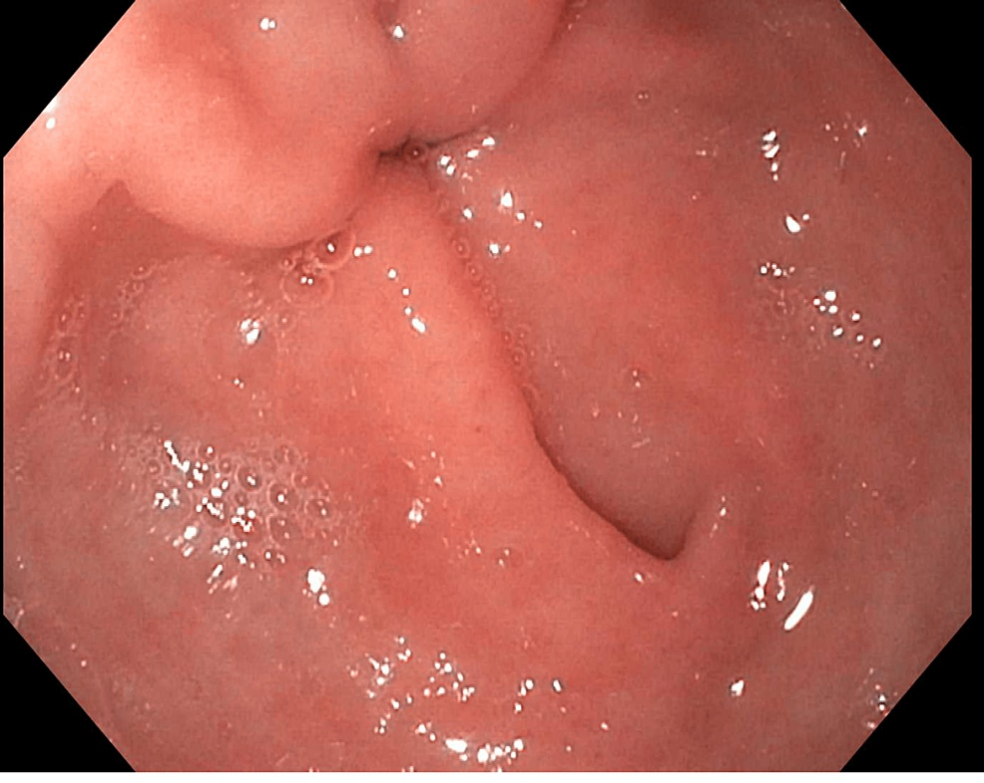
A non-bleeding diverticulum in the gastric antrum

A non-bleeding diverticulum was found in the gastric antrum (Figure 2). Biopsies for H. pylori testing were negative. The patient was treated medically with an oral proton pump inhibitor (PPI), and her symptoms improved upon follow-up appointments.

## Discussion

Gastric diverticulum is an uncommon form of diverticular disease, first described in 1661 by Moebius and in 1774 by Roax [3]. Prevalence of gastric diverticulum varies depending on the diagnostic tool, reaching 0.01-0.11% at EGD, near the gastroesophageal junction in most cases (75%), mainly in the fundus [1,2]. Gastric diverticulum affects males and females equally, most commonly occurring in patients between 20 and 60 years of age. Symptomatic gastric diverticula are rare, and many patients with gastric diverticula remain asymptomatic throughout life. The symptoms that may seek treatment include epigastric pain, dyspepsia, heartburn, gastrointestinal bleeding, and weight loss depending on the size, presence of ectopic tissue, and the location of the gastric diverticulum [4].

Gastric diverticula have been classified into congenital (true) diverticulum, which contains all layers of the gastric wall due to malformation during embryogenesis, and acquired (false) diverticulum, which occurs secondary to gastric pathology, including increased intraluminal pressure and external traction like adhesions. Gastric diverticulum diagnosis is incidental in most cases by radiological images or endoscopy. However, EGD is considered the gold standard to make a diagnosis [2,4]. 

Although most individuals with gastric diverticulum do not require therapy since they are asymptomatic, the management method is mostly determined by the symptoms or consequences of the diverticulum. As long as there is no sign of major complications, such as a stomach ulcer, bleeding, or perforation, conservative care remains the basis of treatment for symptomatic individuals. Medical therapy includes a soft diet, antispasmodics, and PPI, which have been shown to alleviate symptoms in cases with confirmed gastric diverticulum. There are other instances of effective endoscopic therapy of patients with gastric diverticulum that presented with active upper GI hemorrhage in the literature.

Failure of medicinal care, large gastric diverticulum (>4 cm in diameter), or complications like perforation may necessitate surgical therapy, including diverticulum excision with primary repair [2,4,5]. Gastric diverticulum in the antrum is quite uncommon. Clinical presentation and diagnosis need a high level of clinical suspicion, and therapy can be difficult and varies from the diverticulum in other regions of the gastrointestinal system. However, once the gastric diverticulum is diagnosed, routine surveillance is recommended to avoid life-threatening complications, including GI bleeding or gastric perforation.

## Conclusions

Gastric diverticulum is a rare gastrointestinal condition that is characterized by out-pouching of the stomach wall and can result in life-threatening bleeding or perforation. In most cases, the exact site is at the gastroesophageal junction, primarily in the fundus, but the gastric diverticulum can also be present in the antrum. This case sheds light on the importance of considering the gastric diverticulum, even in unusual locations like the antrum, when evaluating patients with gastrointestinal symptoms. 
